# *In vitro, ex vivo*, and *in vivo* evaluation of ophthalmic ointments containing dexamethasone and tobramycin

**DOI:** 10.1016/j.ijpx.2025.100476

**Published:** 2025-12-25

**Authors:** Catheleeya Mekjaruskul, Andre O'Reilly Beringhs, Tuo Meng, Aji Alex Moothedathu Raynold, Qingguo Xu, Matthew Halquist, Bin Qin, Yan Wang, Xiuling Lu

**Affiliations:** a Department of Pharmaceutical Sciences, School of Pharmacy, University of Connecticut, Storrs, CT, USA; b Faculty of Pharmacy, Mahasarakham University, Maha Sarakham 44150, Thailand; c Division of Therapeutic Performance, Office of Research and Standards, Office of Generic Drugs, U.S. Food and Drug Administration, Silver Spring, MD, USA; d Department of Pharmaceutics, School of Pharmacy, Virginia Commonwealth University, Richmond, VA, USA; e Center for Pharmaceutical Engineering, Department of Pediatrics, Department of Biomedical Engineering, Center for Drug Discovery, and Massey Cancer Center, Virginia Commonwealth University, Richmond, VA 23298, USA

**Keywords:** Ophthalmic ointment, *In vitro* release test, *Ex vivo* ocular permeation, *In vivo* ocular pharmacokinetics, Dexamethasone, Tobramycin

## Abstract

This investigation compares *in vitro* release, *ex vivo* release and permeation, and *in vivo* ocular pharmacokinetics to render biologically informed evaluations of ophthalmic semi-solid drug products containing dexamethasone (hydrophobic) and tobramycin (hydrophilic). Both drugs were formulated with three petrolatum matrices (IGI® 320 A, IGI® 386, or Spectrum®) with distinct rheological character and benchmarked against the reference listed drug, Tobradex®. Temperature-sweep rheology revealed that IGI® 386 most closely reproduced the viscoelastic profile of the reference product. USP Apparatus I release testing with surfactant-free medium provided maximal discrimination for dexamethasone (Tobradex® > IGI® 320 A > IGI® 386 > Spectrum®), and rank-order release rates correlated strongly with *ex vivo* corneal permeation and *in vivo* corneal exposure. In contrast, tobramycin required a polysorbate-containing medium to resolve formulation differences *in vitro*, yet those differences did not persist *ex vivo* or *in vivo*, consistent with its rapid dissolution and diffusion, which attenuate matrix effects. The data demonstrate that drug solubility dictates the choice of biorelevant release conditions in petrolatum-based ophthalmic ointments: surfactant-free media capture formulation-dependent release for hydrophobic actives, whereas hydrophilic actives may yield artifactual discrimination when surfactant is present. However, formulations indistinguishable *in vitro* were typically similar in their *in vivo* ocular pharmacokinetics. By integrating tiered models, the framework enhances understanding of critical quality attributes, supports regulatory decision-making, and may help reduce reliance on animal studies, thereby expediting the development of therapeutically equivalent generic ophthalmic ointments.

## Highlights

• *In vitro*, *ex vivo*, and *in vivo* studies evaluated petrolatum-based ophthalmic ointments.
• Surfactant-free IVRT best differentiated hydrophobic dexamethasone, matching *ex vivo* data.
• Surfactant-containing IVRT showed formulation differences not reflected *in vivo*.
• Formulations similar *in vitro* also showed comparable *in vivo* ocular pharmacokinetics.

## Graphical abstract

**Figure:**
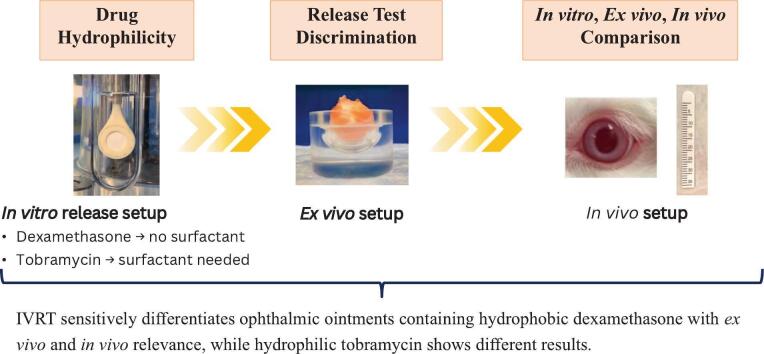

## 1 Introduction

Ophthalmic semi-solid drug products, such as ointments and gels, play a critical role in the treatment of various ocular conditions, including infections, inflammation, and glaucoma. These formulations offer several advantages over liquid preparations, such as prolonged residence time on the ocular surface, reduced dosing frequency, and enhanced patient compliance (Mazet et al., 2020). Despite their widespread use, the evaluation of ophthalmic semi-solid formulations presents significant challenges due to the complex anatomy and physiology of the eye, as well as the unique properties of these dosage forms. Traditionally, the assessment of ophthalmic drug products has relied heavily on *in vivo* studies, which, while providing comprehensive data, are time-consuming, ethically challenging, and expensive. *In vitro* methods, such as *in vitro* drug release testing and permeation studies, offer a more practical alternative but often fail to accurately predict *in vivo* performance due to the lack of physiological relevance (Bao et al., 2018). However, when carefully optimized, *in vitro* release testing can effectively discriminate between the release profiles of different ophthalmic ointments (Bao et al., 2017, Bao et al., 2020; Dong et al., 2018; Mekjaruskul et al., 2023, Mekjaruskul et al., 2024; Patere et al., 2020; Xu et al., 2015b). *Ex vivo* models, using animal tissues, provide a middle ground by incorporating biological barriers, yet they are limited by variability and ethical considerations (Steyn et al., 2025). A key gap in the current literature is the lack of an integrated evaluation framework that combines *in vitro*, *ex vivo*, and *in vivo* methodologies to comprehensively assess and understand sources of performance variability in ophthalmic semi-solid drug products (Renukuntla et al., 2022). Most studies focus on a single aspect, which limits the ability to correlate findings across different models and hampers the development of formulations with optimal therapeutic efficacy. In addition, the drug physicochemical properties especially hydrophilicity directly affect the drug release performance. Evaluation methods and protocol selection may vary, depending on the properties of the active pharmaceutical ingredients (APIs). Some assessments are designed to be highly discriminatory, but the results may or may not correlate closely with *in vivo* performance.

This study aims to address these gaps by investigating a comprehensive assessment framework and systematically comparing the *in vitro* release test (IVRT), *ex vivo*, and *in vivo* evaluation methods for ophthalmic ointments containing both hydrophilic tobramycin and hydrophobic dexamethasone. The discriminatory power, as well as the relevance to the performance prediction *in vivo*, will be evaluated. Additionally, this research will explore the critical quality attributes (CQAs) of selecting active pharmaceutical ingredients (APIs; *i.e.*, dexamethasone and tobramycin) and excipients that influence the performance of semi-solid formulations. The unique contribution of this study lies in its holistic approach to formulation assessment. This framework has the potential to streamline product development and the regulatory assessment process, and ultimately to lead to more ophthalmic therapies.

## 2 Methods

### 2.1 Materials

Dexamethasone was sourced from Medisca® (Medisca Inc., NY, USA). Tobramycin was purchased from Chem-Werth. Inc., USA. Petrolatum was obtained from Spectrum Chemical Manufacturing Co. (NJ, USA), along with two additional variants (IGI® 386 and IGI® 320 A) procured from International Group, Inc. (Canada). Anhydrous chlorobutanol was also acquired from Spectrum Chemical Manufacturing Co. (NJ, USA). The release medium was prepared using sodium bicarbonate (2 mg/mL, Sigma-Aldrich®, MO, USA), sodium chloride (6.7 mg/mL, Merck®, Denmark), calcium chloride (0.08 mg/mL, Sigma-Aldrich®, Japan), with or without surfactant (polysorbate 80, Croda Inc., NJ, USA) at pH 7.4. HPLC-grade acetonitrile was purchased from Fisher Chemical (NJ, USA), and 1.2 μm polyethersulfone membrane filters were supplied by Sterlitech® (WA, USA). The reference ointment was Tobradex® (tobramycin 0.3 % and dexamethasone 0.1 % combination).

### 2.2 High shear processing for semi-solid ointment preparation

Test ointments were prepared in-house using three different petrolatum bases, each formulation incorporating 5 % mineral oil and 0.5 % chlorobutanol, and containing a combination of tobramycin (0.3 %) and dexamethasone (0.1 %) as active pharmaceutical ingredients. The mixing process was carried out using a high-shear mixer (Unguator® e/s, Germany) within an Unguator® jar. The procedure followed the method described by Patere et al. (2018) (Patere et al., 2018), involving sequential mixing at 2100 rpm for 9 min, followed by a 5-min rest period, then mixing at 970 rpm for 1 min, another 5-min rest, and a final mixing step at 810 rpm for 1 min.

### 2.3 Rheological properties

Rheological analysis was conducted using an AR-G2 rheometer (TA Instruments, USA) equipped with a cone and plate geometry setup. The cone had a diameter of 40 mm with a truncation of 30 μm. The rheological properties of Tobradex® and three different petrolatum-based test formulations containing dexamethasone and tobramycin were evaluated through temperature sweep profiling. The measurements were performed over a temperature range from 25 to 45 °C, maintaining a constant strain of 0.05 % and a frequency of 1 Hz. This approach followed the methodology described in a previous study (Mekjaruskul et al., 2023).

### 2.4 Determination of dexamethasone concentration *via* HPLC-PDA

For the analysis, a Shimadzu® HPLC system (Japan) equipped with a PDA detector was employed, following the method previously described (Mekjaruskul et al., 2021). Chromatographic separation was achieved using an Agilent® C18 column (4.6 × 250 mm; 5 μm) as the stationary phase. The mobile phase consisted of a mixture of 40 % acetonitrile and 60 % (*v*/v) water. The system operated at a flow rate of 1 mL/min, with dexamethasone detection set at a wavelength of 241 nm. Each sample injection volume was 20 μL, with a total run time of 10 min per sample. The retention time for dexamethasone was consistently recorded at 7.48 min. All analyses were performed at a controlled temperature of 25 °C. The method exhibited a limit of detection (LOD) of 0.004 μg/mL and a limit of quantification (LOQ) of 0.01 μg/mL, with excellent linearity over the tested concentration range (R^2^ = 0.9999).

### 2.5 Determination of tobramycin concentration *via* HPLC-fluorescence

Tobramycin analysis was conducted based on the protocol established in a previous article (Patere et al., 2018). Briefly, a fluorescamine solution (0.01 % in acetone) was prepared and mixed with the sample. The mixture was incubated in a shaking water bath at room temperature for 20 min to ensure complete reaction. Subsequently, water was added to bring the final volume to 5 mL. After allowing the mixture to stand for an additional 15 min, it was injected into the HPLC system for analysis. A Shimadzu® HPLC system (Japan) equipped with a fluorescence detector was used. Chromatographic separation was achieved using an Agilent® C18 column (2.1 × 100 mm, 3.5 μm). The mobile phase consisted of a 50:50 (*v*/v) mixture of methanol and water, operated in an isocratic mode with a flow rate of 0.3 mL/min. The total run time for each analysis was 10 min, with the tobramycin peak appearing at approximately 1.3 min. All analyses were conducted at a controlled temperature of 25 °C. The method exhibited an LOD of 0.002 μg/mL and an LOQ of 0.007 μg/mL, with excellent linearity over the tested concentration range (R^2^ = 0.9997). Fluorescence detection was performed with excitation at a wavelength of 390 nm and emission at 480 nm.

### 2.6 *In vitro* release testing (IVRT)

IVRT was performed following a methodology previously reported (Mekjaruskul et al., 2024). The experiment utilized the USP Apparatus I equipped with a small volume vessel (Sotax® ATXtend, USA) and a custom-designed, 3D-printed, two-sided adapter made from poly(lactic acid) filament as shown in the supplementary file. Each side of the adapter had a surface area of 3.14 cm^2^, resulting in a total surface area of 6.28 cm^2^. To assemble the system, the first membrane was carefully placed within the bottom cap, followed by positioning the spacer directly on top. A precisely weighed portion of ointment (ranging from 0.5 to 0.6 g) was then evenly distributed over the membrane within the spacer. The second membrane was subsequently placed over the ointment, and the top cap was secured to seal the assembly. This completed adapter setup was then mounted on the holder and attached to the rotating shaft of the USP Apparatus I. The experiment was conducted using 100 mL release medium per vessel, with or without surfactant (0.1 % polysorbate 80), maintained at 37 °C and stirred at 150 rpm. At predetermined intervals, 1 mL of the release medium was sampled and immediately replenished with fresh medium to ensure consistent conditions. The collected samples were centrifuged at 9391 *g* for 10 min, and the supernatant was analyzed using HPLC equipped with a photodiode array (PDA) detector. Cumulative drug release was assessed by plotting the amount released over time, the cumulative release (expressed as μg/cm^2^) *versus* the logarithm of time, or the square root of time for up to one hour. To determine the release rate (K) and correlation coefficient (R^2^), linear regression analysis was performed.

### 2.7 *Ex vivo* drug release using rabbit whole eyes

Freshly excised whole rabbit eyes were obtained from Pel-Freez® Arkansas, LLC (Rogers, AR). The eyes were collected in phosphate-buffered saline (PBS) containing penicillin–streptomycin, amphotericin B, and gentamicin, and shipped on wet ice on the same day of collection to minimize contamination and maintain physiological properties. Prior to use, the eyes were incubated in the release medium for 1 h at 34 °C to ensure equilibration. A single eye was designated for each sampling time point. After the incubation period, 30 mg of test ointment formulated with three different petrolatum bases, as well as the reference ointment (Tobradex®), was applied. Eyes were grouped based on weight to reduce bias, and the ointments were evenly spread onto the central anterior surface of each eye. The application was performed swiftly to minimize the risk of tissue dryness. The release medium (without polysorbate 80) was pre-warmed to 34 °C before being added to the custom-made eye holder (650 μL). Each treated eye was positioned upside down within the holder, with the ointment-covered surface facing the release medium, which was then placed into a polypropylene/polyethylene plastic jar and securely sealed as shown in the supplementary file. The jar was subsequently incubated in a hot air oven set at 34 °C. Samples of the release medium, aqueous humor, and cornea were collected at specified intervals: 5, 10, 15, 30, 45, 60, 120, and 240 min. The residual ointments on the eyes were carefully removed by wiping with tissue paper. The eyes were then soaked in warm phosphate-buffered saline (PBS) containing 0.2 % polysorbate 80 at 40 °C. After soaking, the eyes were wiped again using tissue paper. This cleaning procedure was repeated three times to ensure the complete removal of any residual ointment from the eye surface. Following the cleaning process, the eyes were wrapped in aluminum foil to prevent contamination and stored at −80 °C until further sample collection. The collected release medium samples were filtered using a 0.45 μm polyethersulfone syringe filter from Sterlitech® before injection into the HPLC system.

Prior to cornea separation, the frozen eyes were transferred to a − 20 °C freezer for 5 min to slightly thaw. The cornea was then carefully excised along the limbus, and the aqueous humor was subsequently collected. To prevent cross-contamination between cornea and aqueous humor samples, separate forceps were used for each sample. The excised cornea was cut into small pieces, wrapped in aluminum foil and quickly frozen in liquid nitrogen. The frozen cornea was then immediately smashed with a hammer, as described previously by Meng et al. (2021) (Meng et al., 2021). The smashed cornea tissues were further homogenized in water for 5 min using Bullet Blender® machine (Laboratory Supply Network, NH, USA). The collected aqueous humor was placed directly into a microtube. For both the cornea and aqueous humor samples, 400 μL of methanol (70 % in water) was added. The mixture was thoroughly mixed using a vortex, followed by sonication for 30 min to enhance extraction. After sonication, the samples were centrifuged at 9391 *g* for 10 min to separate the supernatant. The supernatants were carefully collected and filtered through a 0.45 μm polyethersulfone syringe filter from Sterlitech® to ensure the removal of particulate matter. The filtered solution was then directly injected into the HPLC system for analysis. The percent recovery for dexamethasone was 99.16 % in corneal tissue and 95.81 % in aqueous humor, while tobramycin exhibited recoveries of 86.84 % in cornea and 94.71 % in aqueous humor.

The cumulative amounts of dexamethasone and tobramycin released were calculated and plotted against time. Additional plots were generated to show cumulative drug release (μg/cm^2^) as a function of the logarithm of time and the square root of time. Linear regression analysis was used to determine the release kinetics, specifically calculating the release rate (K).

### 2.8 *In vivo* pharmacokinetic studies in rabbit eye compartments

Adult New Zealand White (NZW) rabbits (2–3 kg) with mixed gender were purchased from Robinson Service, Inc. (Mocksville NC). Rabbits were individually housed in the VCU rabbit vivarium with free access of a standard laboratory diet and water. Rabbits were allowed to acclimatize for a minimum of 2 days. Daily visual inspection of appearance and behavior were conducted to ensure the health of the animals and address any signs of distress. A single ocular dose of approximately 50 mg of Tobradex® or test ointments formulated with petrolatum from Spectrum® and IGI® 386 was applied horizontally into the lower conjunctival sac of both eyes of each rabbit (*n* = 3 rabbits and *n* = 6 eyes per group) using a spatula under minimal physical restraint. The lower eyelid was gently moved upwards to spread the dose uniformly over the corneal surface. Rabbits were held for 2 more minutes after the topical application to prevent them from shaking their head or pawing away the doses. Tear samples were collected and measured by using tear strips (TEAR FLO™) gently placed at the medial canthus of the eye to absorb tear fluid at designated time points at 15 min, 30 min, 1 h, 2 h, 3 h, 4 h, 6 h, and 8 h post-administration (total 8 time points) under the minimal physical retrain, as described previously (Meng et al., 2021). After the collection of the tear samples, rabbits were immediately sacrificed by an intravenous injection of sodium pentobarbital (150 mg/kg) after an intramascular injection of 25 mg/kg ketamine and 2.5 mg/kg xylazine. The eyeballs were carefully enucleated and stored at −80 °C until further analysis. The aqueous humor, cornea, conjunctiva, and iris samples (*n* = 6 for each data point) were isolated and processed using the same extraction procedures outlined in the *ex vivo* section.

Quantification of dexamethasone and tobramycin was performed using a previously validated UPLC-MS/MS method as described by Meng et al. (2021) (Meng et al., 2021). UPLC–MS/MS analysis was conducted using an Acquity UPLC I-Class system coupled with a Xevo TQ-S micro triple quadrupole mass spectrometer equipped with an electrospray ionization (ESI) source (Waters, MA, USA). The LC system comprised a binary pump, autosampler, analytical column, and two 6-port switching valves. Chromatographic separation was achieved on a Kinetex PFP (pentafluorophenyl) column (2.1 × 100 mm, 1.7 μm particle size) maintained at 40 °C. The mobile phases consisted of acetonitrile with 0.5 % formic acid (organic phase, B) and water containing 0.14 % trifluoroacetic acid (aqueous phase, A). The injection volume was 20 μL. The total analytical runtime was 6 min. Dexamethasone and tobramycin were detected at retention times of approximately 3.08 and 2.88 min, respectively. Quantitation was performed in multiple reaction monitoring (MRM) mode with optimized compound-dependent parameters: cone voltage and collision energy were set at 32 V and 15 V for dexamethasone (*m*/*z* 393.08 → 355.13) and 30 V and 20 V for tobramycin (m/z 468.20 → 163.10).

The experimental animals were randomly assigned to the three experimental groups. The investigators performing PK analysis were masked to the treatment groups, after which samples were decoded and analyzed independently by at least two investigators. All animal experiments were performed in compliance with the guidelines of the ARVO Statement for the Use of Animals in Ophthalmic and Vision Research, and the protocol was approved by the VCU Animal Care and Use Committee (Protocol # AD10001739).

### 2.9 Statistical analysis

All data are expressed as the mean ± standard deviation (SD). Comparisons between two groups were performed using an independent *t*-test. Comparisons among multiple groups were performed using one-way analysis of variance (ANOVA) followed by Tukey's *post hoc* test to evaluate pairwise differences. A *p*-value of ≤0.05 was considered indicative of statistical significance. All statistical analyses were conducted using GraphPad Prism version 10 (GraphPad Software, Boston, Massachusetts, USA).

## 3 Results

**Fig. 1:**
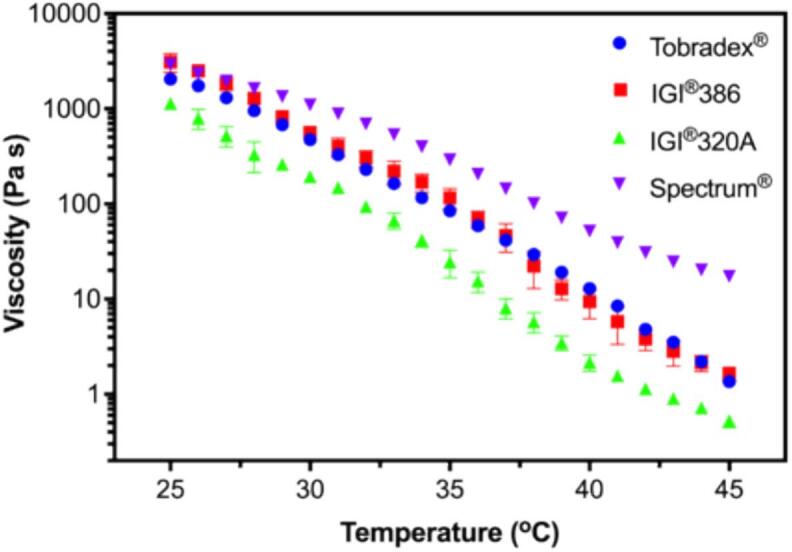
Rheological properties of Tobradex® and three different petrolatum-based formulations containing dexamethasone and tobramycin, presented as temperature sweep profiles.

Temperature sweep profiles revealed distinct rheological behaviors among the petrolatum-based formulations containing dexamethasone and tobramycin (Fig. 1). While all formulations exhibited temperature-dependent changes, the sample prepared with IGI® 386 petrolatum demonstrated rheological characteristics closely resembling those of the reference product, Tobradex®. In contrast, formulations using IGI® 320 and Spectrum® petrolatum showed differing viscoelastic responses over the tested temperature range (25–45 °C), indicating variations in structural consistency and thermal sensitivity. These findings suggest that the choice of petrolatum base could significantly influence the rheological profile of the final ointment formulation.

**Fig. 2:**
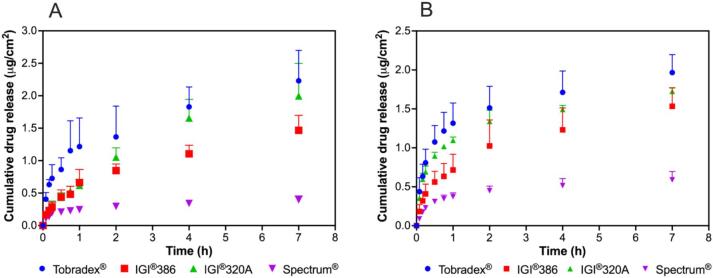
*In vitro* release profiles of dexamethasone from Tobradex® and three different petrolatum-based formulations containing dexamethasone and tobramycin using release media with (A) and without (B) polysorbate 80 (*n* = 3).

**Fig. 3:**
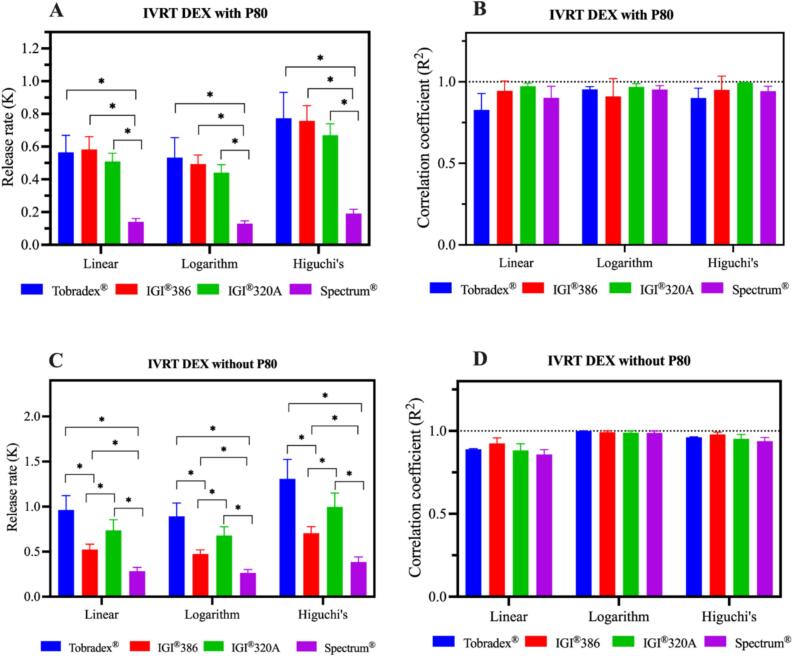
*In vitro* release kinetics and correlation coefficients of dexamethasone from Tobradex® and three different petrolatum-based formulations containing dexamethasone and tobramycin using release media with (A and B) and without (C and D) polysorbate 80 (n = 3).

The *in vitro* release profiles of dexamethasone from Tobradex® and three petrolatum-based formulations were evaluated using release media with and without polysorbate 80 (Fig. 2). Among the test formulations both conditions with and without surfactant, the one containing IGI® 320 petrolatum exhibited a release profile most closely approximating that of the reference product, Tobradex®. In contrast, formulations prepared with IGI® 386 and Spectrum® petrolatum demonstrated reduced and less consistent drug release under both conditions. Based on the release profiles, the surfactant-free condition provided greater discrimination among the test formulations, with more distinct differences in release behavior compared to the condition containing surfactant. The *in vitro* release kinetics and corresponding correlation coefficients are presented in Fig. 3. When polysorbate 80 was incorporated into the release medium, the kinetic parameters across the formulations were generally comparable, exhibiting minimal differentiation despite their distinct rheological properties. Conversely, under surfactant-free conditions, the release kinetics and correlation coefficients revealed more pronounced distinctions between the formulations. Release rates obtained from the three kinetic models, namely linear, logarithmic, and Higuchi's, showed similarly high coefficients of determination with R^2^ values close to 1, and all models consistently demonstrated differentiation among the test formulations. The media without polysorbate 80 demonstrated enhanced sensitivity in differentiating the drug release rates among the tested ointments, effectively reflecting the impact of their rheological differences.

**Fig. 4:**
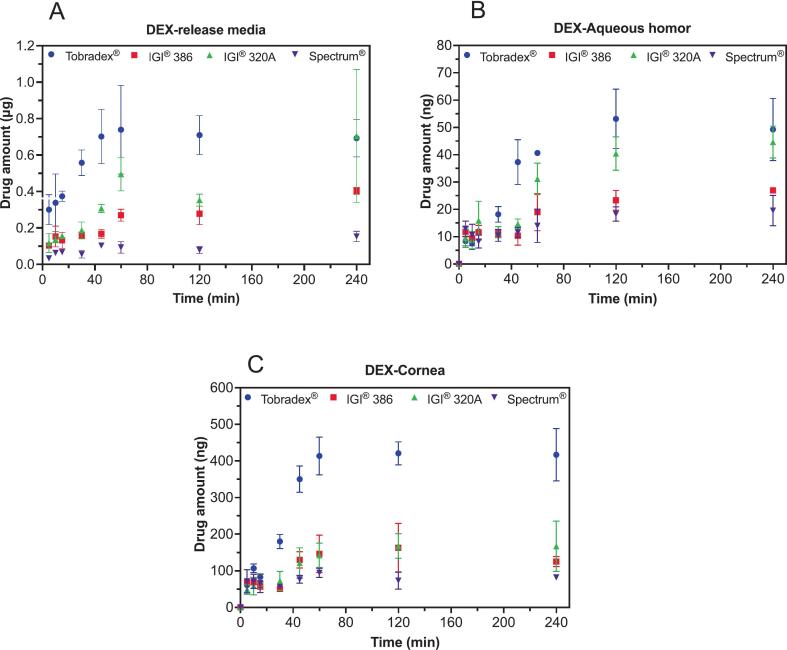
Amount of dexamethasone measured in the release media (A), aqueous humor (B), and cornea (C) following the application of Tobradex® and three different dexamethasone- and tobramycin-containing petrolatum formulations on rabbit eyeballs in an *ex vivo* release study (*n* = 3 eyes).

**Fig. 5:**
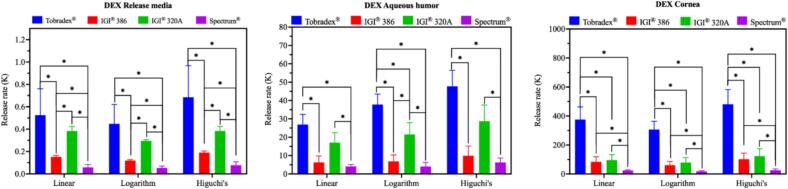
Release kinetics of dexamethasone following the application of Tobradex® and three different petrolatum-based formulations on rabbit eyeballs in an *ex vivo* release study (n = 3 eyes).

The *ex vivo* release of dexamethasone from Tobradex® and three petrolatum-based formulations was evaluated by measuring drug levels in the release medium, aqueous humor, and cornea following application to excised rabbit eyeballs (Fig. 4). In both the release media and aqueous humor (Fig. 4A and B), the dexamethasone concentrations followed the descending order: Tobradex® > IGI® 320 A > IGI® 386 > Spectrum®. For the corneal tissue (Fig. 4C), a different trend was observed, with Tobradex® showing the highest accumulation, followed by IGI® 320 A and IGI® 386 (which performed comparably), and Spectrum® demonstrating the lowest levels. The release kinetics of dexamethasone from Tobradex® and the three petrolatum-based formulations were further evaluated in the *ex vivo* rabbit eye model (Fig. 5). The release rate data, derived from linear regression analysis of cumulative release *versus* time plots, revealed clear differences among the formulations. Consistent with the drug concentration results in the release medium, aqueous humor, and cornea, Tobradex® exhibited the highest release rate, followed by IGI® 320 A, IGI® 386, and Spectrum®. In both the release media and corneal tissue, release kinetics derived from the linear, logarithmic, and Higuchi models consistently distinguished among the test formulations. In the aqueous humor, the linear model did not show statistically significant differences among several formulation pairs, including IGI® 386 *vs* Spectrum®, IGI® 386 *vs* IGI® 320 A, and Tobradex® *vs* IGI® 320 A. In contrast, the logarithmic and Higuchi models demonstrated stronger discriminatory capability, revealing significant differences among these formulations. This contrasts with the results from the release medium and corneal tissues, where all three models consistently differentiated between every formulation pair.

**Fig. 6:**
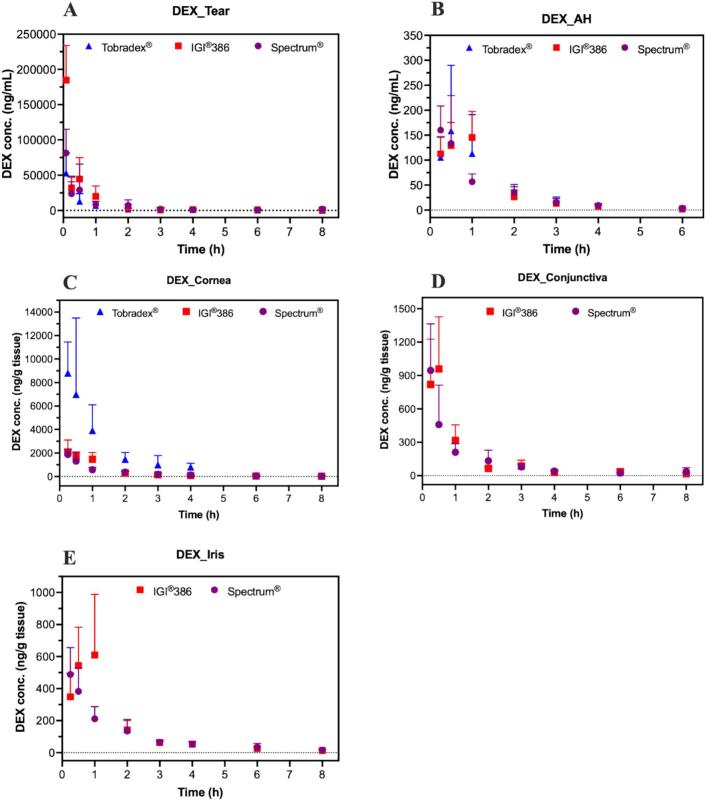
Amount of dexamethasone measured in the tears (A), aqueous humor (B), cornea (C), conjunctiva (D), and iris (E) after the application of Tobradex® and two different formulations containing dexamethasone and tobramycin (Spectrum® and IGI®386) on rabbit eyes (*n* = 6 eyes).

The *in vivo* ocular distribution of dexamethasone was evaluated following the administration of Tobradex®, IGI® 386-based, and Spectrum®-based ointments in rabbit eyes. The pharmacokinetic profiles observed in various ocular tissues are presented in Fig. 6, and the corresponding pharmacokinetic parameters are summarized in Table 1. Dexamethasone concentrations were quantitatively determined in tear fluid, aqueous humor, and cornea after application of all three formulations. In contrast, dexamethasone was detectable in the conjunctiva and iris only after administration of the IGI® 386-based and Spectrum®-based ointments. Dexamethasone levels among the three formulations (Tobradex®, IGI® 386, and Spectrum®) in the tears, aqueous humor, and cornea, and between the two test formulations (IGI® 386 and Spectrum®) in the conjunctiva and iris, were comparable, indicating no significant differences in drug distribution across these ocular tissues. However, a notable difference was observed in the cornea (Fig. 6C), where Tobradex® demonstrated significantly higher dexamethasone concentrations compared to both test formulations. This observation is consistent with the pharmacokinetic findings, as both C_max_ and AUC values in the cornea showed clear differentiation among the three formulations.

**Table 1:** Pharmacokinetic parameters of dexamethasone following administration of Tobradex®, IGI®386, and Spectrum® in ocular tissues on rabbit eyes (n = 6 eyes).

Tissue	Product	Ke (1/h)	T_1/2_ (h)	T_max_ (h)	C_max_ (ng/g)	AUC (h*ng/g)
Tears	Tobradex®	0.642 ± 0.291	1.275 ± 0.573	0.139 ± 0.087	59,400 ± 20,945	27,567 ± 6409
	IGI®386	0.602 ± 0.313	1.340 ± 0.463	0.083 ± 0.000	184,754 ± 49,207^a^	64,498 ± 20,968^a^
	Spectrum®	0.286 ± 0.137	2.836 ± 1.042^a^, ^b^	0.181 ± 0.170	81,120 ± 31,742^b^	49,843 ± 16,274
Aqueous humor	Tobradex®	0.802 ± 0.307	0.959 ± 0.300	0.333 ± 0.129	182.5 ± 117.5	212.6 ± 90.07
	IGI®386	0.554 ± 0.177	1.418 ± 0.642	0.708 ± 0.332^a^	170.1 ± 31.20	226.9 ± 45.54
	Spectrum®	0.530 ± 0.131	1.397 ± 0.447	0.333 ± 0.129^b^	181.3 ± 76.22	202.6 ± 38.56
Cornea	Tobradex®	0.705 ± 0.137	1.014 ± 0.194	0.292 ± 0.102	10,676 ± 4802	11,008 ± 4701
	IGI®386	0.486 ± 0.129	1.513 ± 0.404	0.458 ± 0.292	2474 ± 734.6^a^	2781 ± 487.5^a^
	Spectrum®	0.434 ± 0.208^a^	2.170 ± 1.619	0.333 ± 0.129	1965 ± 514.8^a^	2265 ± 283.5^a^
Conjunctiva	IGI®386	0.333 ± 0.096	2.283 ± 0.882	0.375 ± 0.137	1125 ± 430.6	1069.0 ± 177.6
	Spectrum®	0.319 ± 0.129	2.718 ± 1.773	0.292 ± 0.102	952.2 ± 405.7	1098.0 ± 315.4
Iris	IGI®386	0.357 ± 0.089	2.066 ± 0.598	0.708 ± 0.332	743.2 ± 294.4	1067.0 ± 301.5
	Spectrum®	0.341 ± 0.118	2.271 ± 1.070	0.333 ± 0.129^b^	513.0 ± 173.2	825.2 ± 129.4

a : p < 0.05 when compared to Tobradex®.

b : p < 0.05 when compared to IGI®386.

**Fig. 7:**
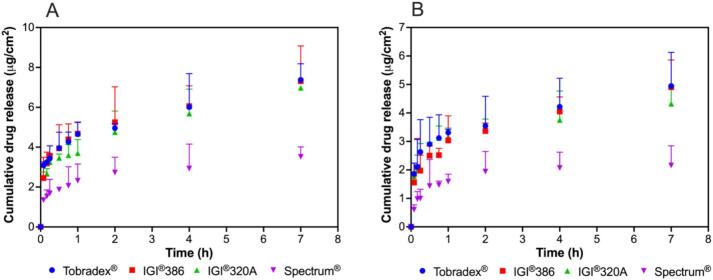
*In vitro* release profiles of tobramycin from Tobradex® and three different petrolatum-based formulations containing dexamethasone and tobramycin using release media with (A) and without (B) polysorbate 80 (*n* = 3 replicates).

**Fig. 8:**
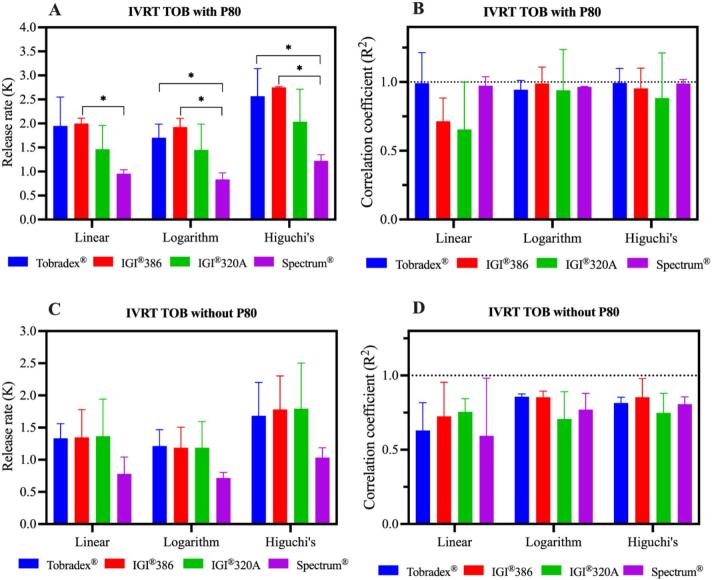
*In vitro* release kinetic and correlation coefficient of tobramycin from Tobradex® and three different petrolatum-based formulations containing dexamethasone and tobramycin using release media with (A and B) and without (C and D) polysorbate 80 (*n* = 3 replicates).

Fig. 7 presents the *in vitro* release profiles of tobramycin from Tobradex® and three petrolatum-based formulations using release media with and without polysorbate 80. In both conditions, the overall release trends were consistent: Tobradex®, IGI® 320 A, and IGI® 386 exhibited higher release rates compared to the Spectrum®-based formulation. Fig. 8 illustrates the *in vitro* release kinetics and corresponding correlation coefficients of tobramycin from Tobradex® and three petrolatum-based formulations using media with and without polysorbate 80. In the absence of polysorbate 80, there were no statistically significant differences in release rates among the formulations in linear, logarithm and Higuchi's models, indicating limited sensitivity of the system for discriminating between ointments with varying petrolatum bases under these conditions. Conversely, in the presence of polysorbate 80, a significant difference in release rate obtained from logarithm and Higuchi's models was observed between Tobradex® and the Spectrum® based formulation and IGI® 386 and Spectrum® based formulation (*p* < 0.05), with Tobradex® and IGI® 386 exhibiting a higher release rate. In contrast, release rates obtained from the linear model showed a significant difference only between IGI® 386 and the Spectrum® based formulation.

**Fig. 9:**
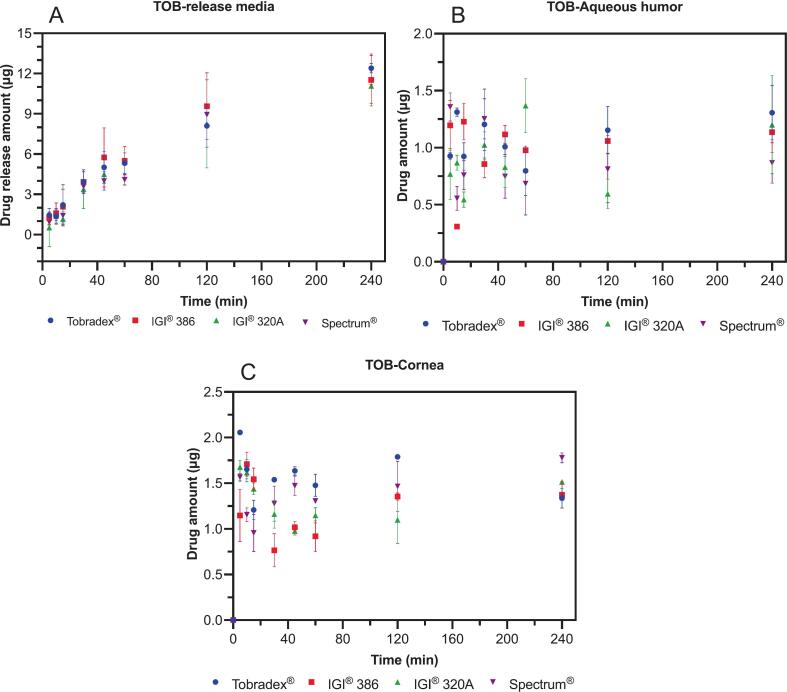
Amount of tobramycin measured in the release media (A), aqueous humor (B), and cornea (C) following the application of Tobradex® and three different dexamethasone- and tobramycin-containing petrolatum formulations on rabbit eyeballs in an *ex vivo* release study (*n* = 3 eyes).

Fig. 9 presents the amount of tobramycin detected in the release medium (A), aqueous humor (B), and cornea (C) following the application of Tobradex® and three petrolatum-based formulations in an *ex vivo* rabbit eye model. Across all three compartments evaluated, no significant differences were observed among the test formulations and the reference product. Tobramycin levels in the release medium, aqueous humor, and corneal tissue were comparable for Tobradex® and the ointments prepared with IGI® 320 A, IGI® 386, and Spectrum® petrolatum bases.

**Fig. 10:**
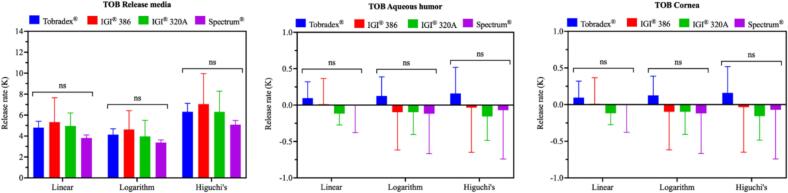
Release kinetics of tobramycin following the application of Tobradex® and three different petrolatum-based formulations on rabbit eyeballs in an *ex vivo* release study (n = 3 eyes).

The release kinetics of tobramycin from Tobradex® and the three petrolatum-based formulations were further assessed in the *ex vivo* rabbit eye model (Fig. 10). Consistent with the drug concentration data shown in the release medium, aqueous humor, and cornea, the release rates of tobramycin were comparable across all formulations in linear, logarithm and Higuchi's models. No significant differences were observed between Tobradex® and the ointments prepared using IGI® 320 A, IGI® 386, or Spectrum® petrolatum bases.

**Fig. 11:**
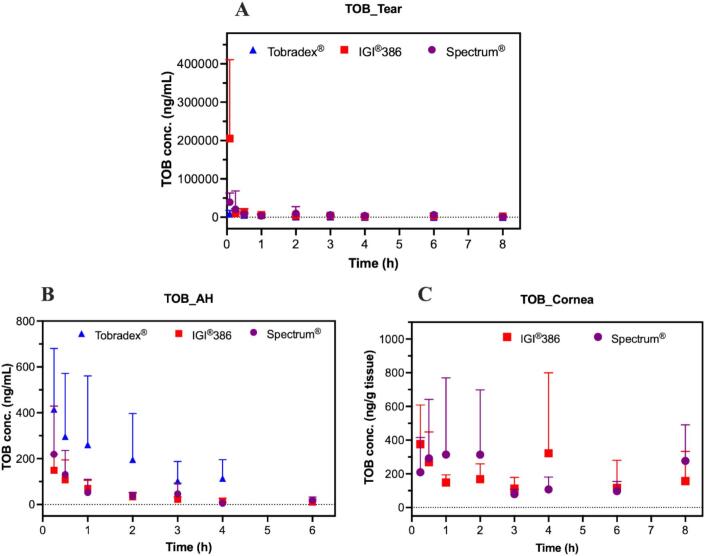
Amount of tobramycin measured in the tears (A), aqueous humor (B), and cornea (C), after the application of Tobradex® and two different formulations containing dexamethasone and tobramycin (Spectrum® and IGI®386) on rabbit eyes (*n* = 6 eyes). Tobramycin was not detectable in cornea for the Tobradex® group.

Following ocular administration of Tobradex® and two petrolatum-based test formulations (IGI® 386 and Spectrum®) in rabbits, tobramycin concentrations were assessed in the tears, aqueous humor, and cornea (Fig. 11). The pharmacokinetic parameters are shown in Table 2. In the tear fluid (Fig. 11A), the IGI® 386 formulation exhibited the highest initial tobramycin levels, followed by Spectrum®, while Tobradex® showed the lowest concentration at the early time point. This observation is consistent with the pharmacokinetic parameters, where the IGI® 386 formulation exhibited the highest Cmax and AUC values in tear fluid, significantly exceeding those of Tobradex®. In contrast, in the aqueous humor (Fig. 11B), Tobradex® demonstrated consistently higher tobramycin concentrations over time compared to both test formulations, indicating superior intraocular penetration. This result aligns with the pharmacokinetic parameters, where Tobradex® exhibited significantly higher Cmax and AUC values in the aqueous humor compared to both test formulations. In the cornea (Fig. 11C), quantifiable tobramycin concentrations were detected only following the administration of IGI® 386 and Spectrum® formulations. The drug levels varied across time points, with no consistent trend or statistically significant differences observed between the two formulations. These findings are consistent with the pharmacokinetic parameter results, which similarly showed no significant difference between IGI® 386 and Spectrum® in the cornea.

**Table 2:** Pharmacokinetic parameters of tobramycin following administration of Tobradex®, IGI®386, and Spectrum® in ocular tissues on rabbit eyes (n = 6 eyes).

Tissue	Product	Ke (1/h)	T_1/2_ (h)	T_max_ (h)	C_max_ (ng/g)	AUC (h*ng/g)
Tears	Tobradex®	0.519 ± 0.157	1.467 ± 0.520	0.222 ± 0.068	19,222 ± 5807	14,538 ± 3077
	IGI®386	0.238 ± 0.182	5.784 ± 5.466	0.083 ± 0.000	205,003 ± 206,019^a^^b^	86,672 ± 66,842^a^
	Spectrum®	0.359 ± 0.357	3.000 ± 1.411	0.500 ± 0.753	53,372 ± 35,554	53,625 ± 23,032
Aqueous humor	Tobradex®	0.806 ± 0.429	1.037 ± 0.460	1.000 ± 0.822	540.1 ± 183.2	876.1 ± 292.6
	IGI®386	0.449 ± 0.110	1.640 ± 0.477	0.417 ± 0.303	171.6 ± 56.18^a^	226.2 ± 39.2^a^
	Spectrum®	0.328 ± 0.197^a^	3.436 ± 2.817	0.292 ± 0.102	253.0 ± 199.4^a^	358.2 ± 162.4^a^
Cornea	IGI®386	0.124 ± 0.095	5.993 ± 2.957	2.208 ± 3.199	619.6 ± 359.3	3509 ± 4109
	Spectrum®	0.287 ± 0.000	2.417 ± 0.000	2.417 ± 2.800	676.9 ± 448.5	1217 ± 0.000

a : p < 0.05 when compared to Tobradex®.

b : p < 0.05 when compared to IGI®386.

## 4 Discussion

The IVRT method developed is intended to provide sensitive discrimination for evaluating formulations and generic preparations. As such, *in vitro* evaluations based on this IVRT can be overly sensitive, as the *in vivo* use may show no differences. However, the similarity confirmed through IVRT would provide confidence for consistent similarity *in vivo*. The *ex vivo* test medium was designed to closely mimic physiological conditions and to serve as a translational bridge between *in vitro* and *in vivo* models. Since the natural tear fluid does not contain surfactants, the *ex vivo* model was conducted under surfactant-free conditions to better reflect the *in vivo* ocular environment and to capture the intrinsic release and permeation characteristics of the formulations.

For hydrophobic drugs, with dexamethasone as a representative poorly water-soluble corticosteroid, the release medium without polysorbate 80 demonstrated greater sensitivity in distinguishing among petrolatum-based formulations. This increased discriminative ability is likely due to the limited aqueous solubility of dexamethasone, which makes formulation-dependent factors such as rheology and ointment microstructure more influential in controlling release. The *in vitro* release ranking (Tobradex® > IGI® 320 A > IGI® 386 > Spectrum®) observed under surfactant-free conditions was consistent with general trends seen in *ex vivo* corneal permeation and *in vivo* corneal exposure. These findings parallel previous work with loteprednol etabonate, another hydrophobic corticosteroid (Uner et al., 2023), where *in vitro* release rates showed a strong linear correlation with *ex vivo* transcorneal flux across four Q1/Q2 equivalent ointments (R^2^ > 0.98) (Bao et al., 2018). Similar correlations have also been observed for loteprednol etabonate ophthalmic gels, further supporting the potential utility of *in vitro*–*ex vivo* comparisons in evaluating formulation performance for poorly water-soluble ocular drugs (Priya et al., 2023). The findings in the current study suggest that surfactant-free IVRT can provide a more formulation-sensitive assessment for hydrophobic actives and may serve as a useful tool for differentiating performance among complex oleaginous ophthalmic formulations as summarized in Table 3.

**Table 3:** Summary of comparative findings across *in vitro*, *ex vivo*, and *in vivo* methods for evaluating dexamethasone ophthalmic ointments.

Method	Dexamethasone	Sensitivity for Differentiation
IVRT in surfactant-free medium	Release rates: Tobradex® > IGI® 320 A > IGI® 386 > Spectrum® at *p* < 0.05. Release amounts are consistent with the release rates.	Very Sensitive
IVRT in surfactant-containing medium	Release rates: Tobradex® > Spectrum® at p < 0.05. Release amounts are consistent with the release rates.	Sensitive
*Ex vivo* study	⁃ Release rates in release media and aqueous humor: Tobradex® > IGI® 320 A > IGI® 386 > Spectrum® ⁃ Release rates in cornea: Tobradex® > IGI®320 A = IGI®386 > Spectrum®	Sensitive; more consistent with *in vivo* results and able to reflect variations observed *in vivo*.
*In vivo* study in rabbits	⁃ No difference between IGI® 320 A and IGI® 386 ⁃ Tears AUC: IGI® 386 > Tobradex® ⁃ Cornea AUC: Tobradex® > IGI® 386 & Spectrum®	High variability was observed *in vivo*.

In contrast, for hydrophilic drug, with tobramycin as a representative highly water-soluble aminoglycoside, the inclusion of polysorbate 80 in the release media improved the *in vitro* resolution of the formulation differences, consistent with previous findings demonstrating that the additional of polysorbate 80 enhanced tobramycin release from the ointments (Patere et al., 2020). However, these *in vitro* differences were not mirrored *ex vivo* or *in vivo*, where tissue concentrations across formulations were largely superimposable, suggesting rapid dissolution and diffusion dynamics minimized formulation-dependent effects in biological environments. A prior study on acyclovir, another hydrophilic compound (Mady et al., 2023), similarly observed that while IVRT indicated limited drug release*, in vitro* transcorneal drug permeation study across rabbit cornea revealed significantly greater corneal permeation within the same time frame (Al-Ghabeish et al., 2015; Xu et al., 2015b). This discrepancy may be attributed to the lipophilic nature of petrolatum in the oleaginous base, which likely facilitated interaction with the lipophilic corneal epithelium and reduced the barrier to drug diffusion. Additionally, such interactions may have expanded the transient boundary layer at the formulation–epithelium interface, making more drug available for dissolution and subsequent permeation (Al-Ghabeish et al., 2015). It is important to note that this transient boundary hypothesis is specifically applicable to oleaginous-base ointments containing solid drug particles, such as hydrophilic drugs dispersed within petrolatum (Xu et al., 2015a). Tobramycin release is primarily limited by water accessibility to the drug within the ointment layer. It is likely that the surfactant disrupts the hydrophobic petrolatum surface layer, enhancing water influx and thereby increasing drug release. Differences in petrolatum viscosity may influence the extent of interaction with surfactants, resulting in varying degrees of drug accessibility within the inner layer. Consequently, the presence of surfactants in the release medium may better distinguish formulations with different viscosities. Since tobramycin dissolves almost instantaneously in the tear film, its ocular disposition is governed more by precorneal clearance and tissue permeability than by matrix rheology. Therefore, the apparent *in vitro* differences observed with surfactant are unlikely to consistently predict *in vivo* performance as summarized in Table 4. In contrast, dexamethasone demonstrated formulation-dependent release characteristics, where matrix rheology and ointment microstructure significantly influenced its release and absorption. These findings highlight that IVRT conditions—particularly those containing surfactants—may vary in differentiating capability depending on the solubility of the drug and the formulation matrix. Complementary use of *ex vivo* and *in vivo* models can help provide a more complete understanding of drug release characteristics from petrolatum-based ointments, especially for highly soluble actives.

**Table 4:** Summary of comparative findings across *in vitro*, *ex vivo*, and *in vivo* methods for evaluating tobramycin ophthalmic ointments.

Method	Tobramycin	Sensitivity for Differentiation
IVRT in surfactant-free medium	Release rates: no significant differences. Spectrum® has lower release amounts.	Not sensitive but similar to *ex vivo* results
IVRT in surfactant-containing medium	Release rates: Tobradex® > Spectrum® and IGI® 386 > Spectrum® at p < 0.05. Spectrum® has lower release amounts.	Sensitive for differentiation but not consistent with *in vivo*
*Ex vivo* study	Tobradex® & all three ointments prepared with three petrolatum sources were similar.	Release was fast with no difference between preparations, due to high solubility of tobramycin
*In vivo* study in rabbits	▪ No differences between the prepared formulations. ▪ Tears AUC: IGI® 386 > Tobradex® ▪ Aqueous AUC: Tobradex® > preparations	Higher viscosity might facilitate the longer retention and so the drug contents of IGI® 386 in tears were higher.

These results underscore the importance of tailoring IVRT conditions to the solubility profile of the active ingredient: surfactant-free media capture formulation-dependent release for hydrophobic drugs, whereas surfactant-containing systems may over-predict differences for hydrophilic drugs that are not evident biologically. Taken together, the tiered framework (IVRT → whole-eye *ex vivo* → targeted *in vivo* pharmacokinetics) may provide a mechanistically coherent continuum for informing formulation development, helping to contextualize laboratory data to clinical surrogates, and potentially reducing animal use on the long term.

Additionally, the observed longer T_max_ (∼2 h) of tobramycin in the cornea, compared to the shorter T_max_ of dexamethasone (less than 0.5 h), may reflect differences in drug diffusion and retention behavior. The hydrophilic nature of tobramycin limits its ability to partition into the lipophilic corneal epithelium, potentially delaying peak tissue levels despite its rapid dissolution in tear fluid. In contrast, dexamethasone, being lipophilic, more readily diffuses across the epithelium, resulting in a faster T_max_. These kinetic differences further support the role of drug physicochemical properties, such as solubility and lipophilicity, in governing ocular tissue distribution, and highlight the importance of considering T_max_ alongside C_max_ and AUC when interpreting *in vivo* performance.

Notwithstanding these strengths, the study has limitations. The *in vivo* evaluation was confined to a single species, dose, and administration technique; extrapolation to human ocular physiology therefore warrants caution. Moreover, only petrolatum-based matrices were examined, leaving open questions regarding alternative structuring systems. Future work should incorporate dynamic tear-flow models, assess mucoadhesive properties, and employ advanced imaging (*e.g.*, optical coherence tomography, fluorophotometry) to capture spatiotemporal drug distribution. Expanding the framework to additional therapeutic classes, such as macrolides and prostaglandin analogs, will further test its applicability and may help inform the development of regulatory standards for complex ophthalmic generics.

## 5 Conclusions

The present work proposes a tiered evaluation paradigm—*in vitro* release, whole-eye *ex vivo*, and *in vivo* pharmacokinetics in eye compartments—that enables mechanistically grounded assessment of ophthalmic semi-solid drug products. Discriminatory performance of IVRT was governed by the interplay between drug solubility, water/drug diffusivity, and medium composition: surfactant-free media accurately ranked petrolatum-based formulations containing the hydrophobic corticosteroid dexamethasone and the resulting hierarchy generally aligned with *ex vivo* corneal uptake and *in vivo* corneal exposure. Conversely, for the highly water-soluble aminoglycoside tobramycin, surfactant-augmented media exacerbated formulation differences that were not observed *in vivo*, highlighting the risk of over-interpretation when test conditions are not bio-informed. Notably, formulations that appeared indistinguishable under *in vitro* conditions exhibited broadly similar *in vivo* ocular pharmacokinetics. By providing a coherent, stepwise approach to correlate critical quality attributes with ocular performance, the framework can streamline formulation development, inform generic product evaluation, and potentially reduce reliance on *in vivo* bioequivalence studies, ultimately supporting patient access to safe and effective generic ophthalmic ointments.

## CRediT authorship contribution statement

**Catheleeya Mekjaruskul:** Writing – original draft, Methodology, Formal analysis, Data curation, Conceptualization. **Andre O'Reilly Beringhs:** Writing – original draft, Formal analysis, Data curation, Conceptualization. **Tuo Meng:** Formal analysis, Data curation. **Aji Alex Moothedathu Raynold:** Formal analysis, Data curation. **Qingguo Xu:** Writing – review & editing, Supervision, Methodology, Investigation. **Matthew Halquist:** Methodology, Formal analysis. **Bin Qin:** Writing – review & editing, Supervision, Project administration. **Yan Wang:** Writing – review & editing, Supervision, Project administration, Conceptualization. **Xiuling Lu:** Writing – review & editing, Supervision, Funding acquisition, Conceptualization.

## Funding

This study was funded by Contract HHSF223201810114C from the 10.13039/100000038U.S. Food and Drug Administration. The opinions and conclusions presented in this article are solely those of the authors and do not represent the official policies or positions of the U.S. Food and Drug Administration or the U.S. Department of Health and Human Services. Furthermore, any reference to specific trade names, commercial practices, or organizations does not imply endorsement by the United States Government.

## Declaration of competing interest

The authors declare a potential conflict of interest associated with the content of this article. A device featured in this study—the two-sided semisolid adapter—has been licensed to SOTAX Corporation (2400 Computer Drive, Westborough, MA 01581, USA). Dr. Xiuling Lu, one of the authors, may receive financial compensation or royalties from its sale or commercial application under this licensing arrangement. However, all aspects of the study, including its design, data acquisition, analysis, and interpretation, were conducted with scientific independence and without external influence. The authors affirm that the findings and conclusions presented are grounded in objective, methodologically sound research.

## Data Availability

Data will be made available on request.

## Appendix A Supplementary data

Supplementary material: Experimental setups used in the study. Image 1
