# Supplementary material for: In vitro, ex vivo, and in vivo evaluation of ophthalmic ointments containing dexamethasone and tobramycin
Source: Int J Pharm X. 2025 Dec 25;11:100476. doi: 10.1016/j.ijpx.2025.100476 (PMC12808560; doi:10.1016/j.ijpx.2025.100476)
Supplement: Supplementary file 1 — Supplementary material: Experimental setups used in the study. [file mmc1.docx]

**Supplementary Materials**

***In Vitro, Ex Vivo*, and *In Vivo* Evaluation of Ophthalmic Ointments Containing Dexamethasone and Tobramycin**

Catheleeya Mekjaruskul^a,b^*^#^*, André O’Reilly Beringhs^c^*^#^*, Tuo Meng^d^, Aji Alex Moothedathu Raynold^d^, Qingguo Xu^d,e^, Matthew Halquist^d^, Bin Qin^c^, Yan Wang^c^, Xiuling Lu^a*^


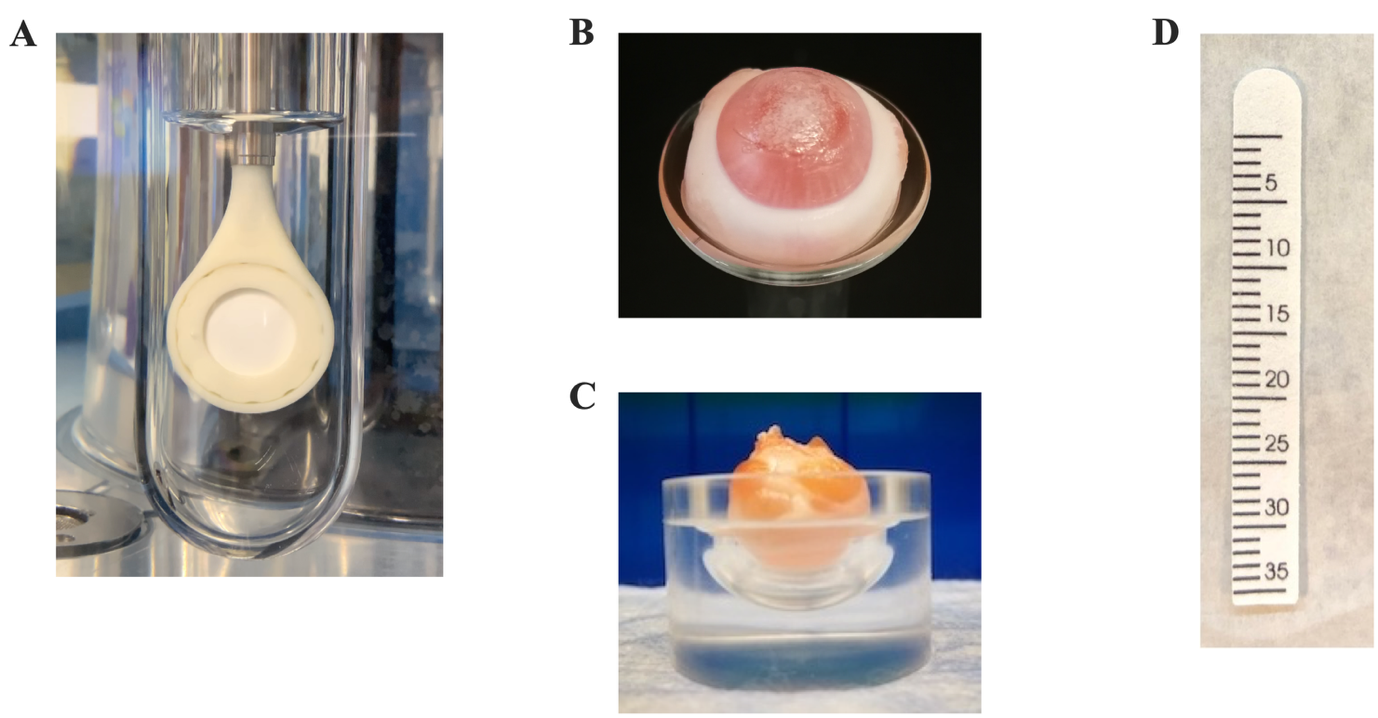


**S. Figure 1**

Experimental setups used in the study. (A) USP Apparatus I equipped with a small-volume vessel and a custom-designed, 3D-printed, two-sided adapter for *in vitro* release testing. (B) Rabbit eyeball used in *ex vivo* drug permeation testing. (C) Eye holder used to position the treated eye upside down during *ex vivo* testing. And (D) Tear collection strip used to collect tear samples during *in vivo* pharmacokinetic studies.
